# A visual interactive analytic tool for filtering and summarizing large health data sets coded with hierarchical terminologies (VIADS)

**DOI:** 10.1186/s12911-019-0750-y

**Published:** 2019-02-14

**Authors:** Xia Jing, Matthew Emerson, David Masters, Matthew Brooks, Jacob Buskirk, Nasseef Abukamail, Chang Liu, James J. Cimino, Jay Shubrook, Sonsoles De Lacalle, Yuchun Zhou, Vimla L. Patel

**Affiliations:** 1 College of Health Science and Professions, Grover Center W357, Ohio University, Athens, OH 45701 USA; 2 Russ College of Engineering and Technology, Ohio University, Athens, OH USA; 3 Informatics Institute, School of Medicine, University of Alabama, Birmingham, AL USA; 4 College of Osteopathic Medicine, Touro University, Vallejo, California, USA; 5 College of Osteopathic Medicine, Ohio University, Athens, OH USA; 6 Patton College of Education, Ohio University, Athens, OH USA; 7 The New York Academy of Medicine, New York, NY USA

**Keywords:** Data analytic tool, Data set filtering, Hierarchical terminology, Visualization, Human comprehension

## Abstract

**Background:**

Vast volumes of data, coded through hierarchical terminologies (e.g., International Classification of Diseases, Tenth Revision–Clinical Modification [ICD10-CM], Medical Subject Headings [MeSH]), are generated routinely in electronic health record systems and medical literature databases. Although graphic representations can help to augment human understanding of such data sets, a graph with hundreds or thousands of nodes challenges human comprehension. To improve comprehension, new tools are needed to extract the overviews of such data sets. We aim to develop a visual interactive analytic tool for filtering and summarizing large health data sets coded with hierarchical terminologies (VIADS) as an online, and publicly accessible tool. The ultimate goals are to filter, summarize the health data sets, extract insights, compare and highlight the differences between various health data sets by using VIADS. The results generated from VIADS can be utilized as data-driven evidence to facilitate clinicians, clinical researchers, and health care administrators to make more informed clinical, research, and administrative decisions. We utilized the following tools and the development environments to develop VIADS: Django, Python, JavaScript, Vis.js, Graph.js, JQuery, Plotly, Chart.js, Unittest, R, and MySQL.

**Results:**

VIADS was developed successfully and the beta version is accessible publicly. In this paper, we introduce the architecture design, development, and functionalities of VIADS. VIADS includes six modules: user account management module, data sets validation module, data analytic module, data visualization module, terminology module, dashboard. Currently, VIADS supports health data sets coded by ICD-9, ICD-10, and MeSH. We also present the visualization improvement provided by VIADS in regard to interactive features (e.g., zoom in and out, customization of graph layout, expanded information of nodes, 3D plots) and efficient screen space usage.

**Conclusions:**

VIADS meets the design objectives and can be used to filter, summarize, compare, highlight and visualize large health data sets that coded by hierarchical terminologies, such as ICD-9, ICD-10 and MeSH. Our further usability and utility studies will provide more details about how the end users are using VIADS to facilitate their clinical, research or health administrative decision making.

## Background

Hierarchical terminologies, such as the International Classification of Diseases 9th Revision-Clinical Modification (ICD9-CM) [1], ICD10-CM [2], SNOMED CT [3], Logical Observation Identifiers Names and Codes (LOINC) [4], RxNorm [5], and Gene Ontology (GO) [6], have been used in biomedicine for a long time. Enormous volumes of data, coded through hierarchical terminologies, are generated continuously within electronic health record (EHR) systems, in biomedical literature databases (such as Medical Subject Headings [MeSH] [7] in PubMed), and in other sources of information. The EHR adoption rate in the United States had reached 96% [8] in hospitals and 87% [9] in office-based physician practices by 2015, and these rates are continually increasing. EHRs contain a wealth of clinical data represented in hierarchical structures, and the large and continually generated data sets in EHRs can be used to explore new patterns or to reveal unknown facts about disease and health as well as to optimize care delivery operations. The large size of these health data sets and their complex terminology structure make them difficult for clinicians, clinical researchers, or administrators to understand. Further processing and analysis of these health data sets are needed to make them manageable and comprehensible.

One way to analyze the large health data sets coded by hierarchical terminologies is to use graphical representations to demonstrate the relationships among the terms. Graphs have long been used to represent hierarchical information as a means to facilitate comprehension. Notably, the human brain can comprehend graphs when the data set is not too large, empirically, and contains no more than 120 nodes. A graph with thousands of nodes is challenging for both the presentation and comprehension of the information. The sizes of the terminology range from dozens of thousands (e.g., ICD9-CM) to over a million terms (e.g., SNOMED CT). A visualization of that scale would not be comprehensive to the human brain. Further filtering to reduce the sizes of the data sets is necessary. Neol [10] and Homer [11] each developed methodologies to reduce the complexity of the graphs and to aggregate hierarchical structures to analyze information security networks. Gu [12, 13] developed methodologies to partition large terminologies for further usage. None, however, developed methods specifically to provide a summary or comparison of data sets based on the semantic relationships of the terminologies and analytic results. Our group has developed the core algorithms to aggregate, filter, summarize, and compare large data sets coded by hierarchical terminologies [14] and methods to set thresholds, demonstrated case studies [15], and published the preliminary results of the comparison of two solutions to develop an online tool for the algorithms [16]. To make the algorithms more accessible to broader audiences, we believe that it is necessary to develop an online, publicly accessible tool to implement the algorithms and to facilitate the decision-making process by clinicians, clinical researchers, and healthcare administrators more conveniently.

We used the software, Graphviz [17] during the algorithm development and case study demonstration. Graphviz is a powerful tool that provides many different types of graphs, such as hierarchical (i.e., dot). Graphviz, however, cannot typically be used by users who lack programming skills. Further, the hierarchical graphs created by Graphviz are static and are not efficient in terms of the use of available screen space. We are developing a Web version of a visual interactive analysis tool for filtering and summarizing large data sets coded with hierarchical terminologies (VIADS) to make the algorithms publicly accessible. VIADS provides more interactive visual features to facilitate users’ comprehension of the data sets and can be used for educational and research purposes at no cost.

## Implementation

### Design modules in VIADS

**Fig. 1:**
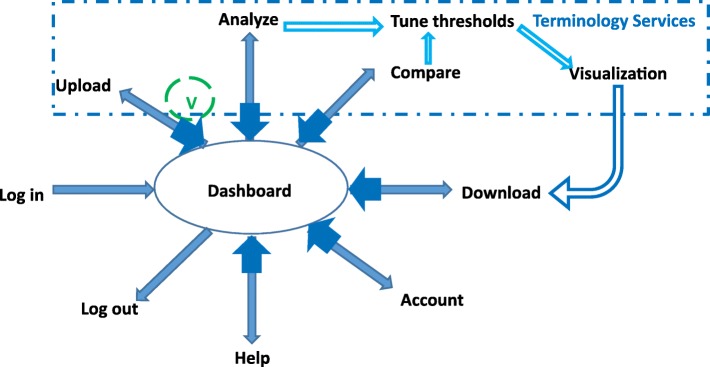
VIADS architecture design and relationships among different modules (V refers to the validation and preparation module; a single arrow indicates a user can move toward one direction; a double arrow indicates a user can move both directions)

There are six modules in VIADS: user management, data sets validation and preparation, dashboard, terminology, analysis, and visualization. Figure 1 presents the architecture design of VIADS and the relationships among different modules.

Figure 1 shows that the dashboard is a central hub for the different modules. Almost all of the data can flow between each module and dashboard in both directions, which are indicated by double arrows in Fig. 1. The design provides more flexibility for users among modules, i.e., a user can always return to the dashboard before access to another module and saves clicks, as compared to a linear design, from start to end.

Users will have to upload a data set first to use VIADS. The validation and preparation module will be initiated as soon as the upload completes. For any new data sets, the validation and preparation module will have to proceed before any analysis or visualization of the data set. During the validation, the user will be presented with the summary profile of the uploaded data sets and options to revise or delete any error records. VIADS can analyze and visualize validated data sets. Tuning of the thresholds is a critical step during data analysis prior to visualization. The comparison is part of the analytic module, and it is separated out in Fig. 1 due to its importance in the tool. All of the results that have been visualized can be downloaded for future use.

Log-in, log-out, and account modules are applied only to registered users. All other modules are applied to both guest users and registered users. Table 1 provides a summary of the main difference between the two groups.

**Table 1:** Usage comparison between guest users and registered users in VIADS

	Guest Users	Registered Users
Register	N/A	√
Log in	N/A	√
Upload test data sets	√	√
Select algorithm	√	√
Tune thresholds to filter data sets	√	√
Generate summary views >= thresholds	√	√
Generate comparison views >= thresholds	√	√
Export analytic results	√	√
Test data sets and analytic results	Deleted at the end of the visit or deleted >= 24 h	Saved on the server side for at least 5 years (free); can be deleted by users manually
Access to the saved results	N/A	√ + VIADS maintenance personnel
Log out	N/A	√
Web site visit log records	Kept	Kept

The terminology module serves mainly the validation and preparation, analytic, and visualization modules. Terminology services provide the default hierarchical structures for supported terminologies to ensure the accurate analysis and visualization of the data sets that they code. Currently, VIADS supports ICD9, ICD10, and MeSH. Therefore, VIADS is able to present data sets coded in both mono-hierarchies and poly-hierarchies. The acceptable data sets of VIADS have to meet two criteria: the data need to be coded using a hierarchical coding system (e.g., ICD9-CM), and frequencies need to be available for each code. These criteria also guide the implementation of the validation and preparation module. Table 2 presents the acceptable formats and sizes of the uploaded data sets.

**Table 2:** Acceptable data sets’ format and size in VIADS

Data set	Node ID (code)	Usage frequency
ICD9-CM	250.00	1223
	401.9	25,567
	… …	…
ICD10-CM	E11.9	4559
	I10	3000
	… …	…
MeSH	A0087342	26,460
	A0021563	24,459
	… …	…
Acceptable data set size for VIADS	Patient counts	> = 100
	Event counts	> = 1000

### Development of VIADS

The development proceeded through various stages: feasibility explorations, comparison studies, local development of individual modules, web migration and module combination, internal tests, and revisions. The main tools and the development environments that we utilized in developing VIADS include Django, Python, JavaScript, Vis.js, Graph.js, JQuery, Plotly, Chart.js, Unittest, R, and MySQL. Django was used to process server-side tasks and the framework of the website. Most of the program was coded using plain JavaScript and Python, including the dashboard, validation and preparation module, user management, terminology, and most of the analysis and visualization modules. To generate the graphs, VIADS uses VIS.js, a Graphviz derivative written in JavaScript. For preview graphs, we used Plotly and Graph.js. VIADS also calls statistical analysis functions from R during the execution of certain algorithms in the analysis module. MySQL is mainly used to manage user accounts.

## Results

### Algorithms implemented in VIADS

VIADS can be utilized to generate a new dimensional perspective on data sets that, in turn, can be utilized to facilitate more informed administrative decisions (e.g., to allocate resources), research decisions (e.g., to validate or deny hypotheses), or clinical decisions (e.g., to select similar medications based on analysis of aggregated data sets). VIADS can be utilized to conduct secondary data analysis, aggregating, filtering, visualization, hypotheses generation, and validation, including exploring new patterns, new facts, or relationships, by looking at the aggregated effects within the data sets. The summarizing, filtering, comparing, and visualizing of capabilities provided by VIADS cannot be accomplished by any single existing tool. Table 3 provides a summary of the algorithms that we implemented in VIADS and their usage examples.

**Table 3:** Algorithms implemented in VIADS with examples of their usage

Filter	Definition	Usage example
NC (node counts)	NC = usage frequency of a node (ICD code or MeSH term)	Displaying a summary view: the most frequently used MeSH terms and their ancestors in 2011
CC (class counts)	CCnode = NCdescendant1 + NCdescendant2 + NCdescendant 3…	Displaying a summary view: the most frequently used ICD9-CM codes in 2011 in a selected institution
Ratio	Ratio = CCchild node/ CCparent node	Identifying the largest MeSH contributors to upper-level MeSH terms and their ancestors in 2011
Top nodes	Top NC nodes (numbers) Top CC nodes (numbers) Top NC% (percentages) Top CC% (percentages)	Displaying the top 50 or (top 5%) of ICD9-CM codes that have the highest NC (or CC) in 2018 in a selected institution. This algorithm can show the most important nodes in a date set
Systematic comparison (data set1 vs data set2)	CCnode1 vs CCnode1; CCnode2 vs CCnode2; CCnode3 vs CCnode3; … …	Displaying a comparison view: the most significant different ICD9-CM codes between pioglitazone(data set1) and rosiglitazone (data set2) groups after systematical comparison
Combination	NC + Ratio	Displaying a summary view: the most frequently used MeSH terms and the largest MeSH contributors and their ancestors in 2011
	CC + Ratio	Displaying a summary view: the most frequently used ICD9-CM codes and the largest ICD9-CM contributors and their ancestors in 2011

### Analytic module workflow in VIADS

**Fig. 2:**
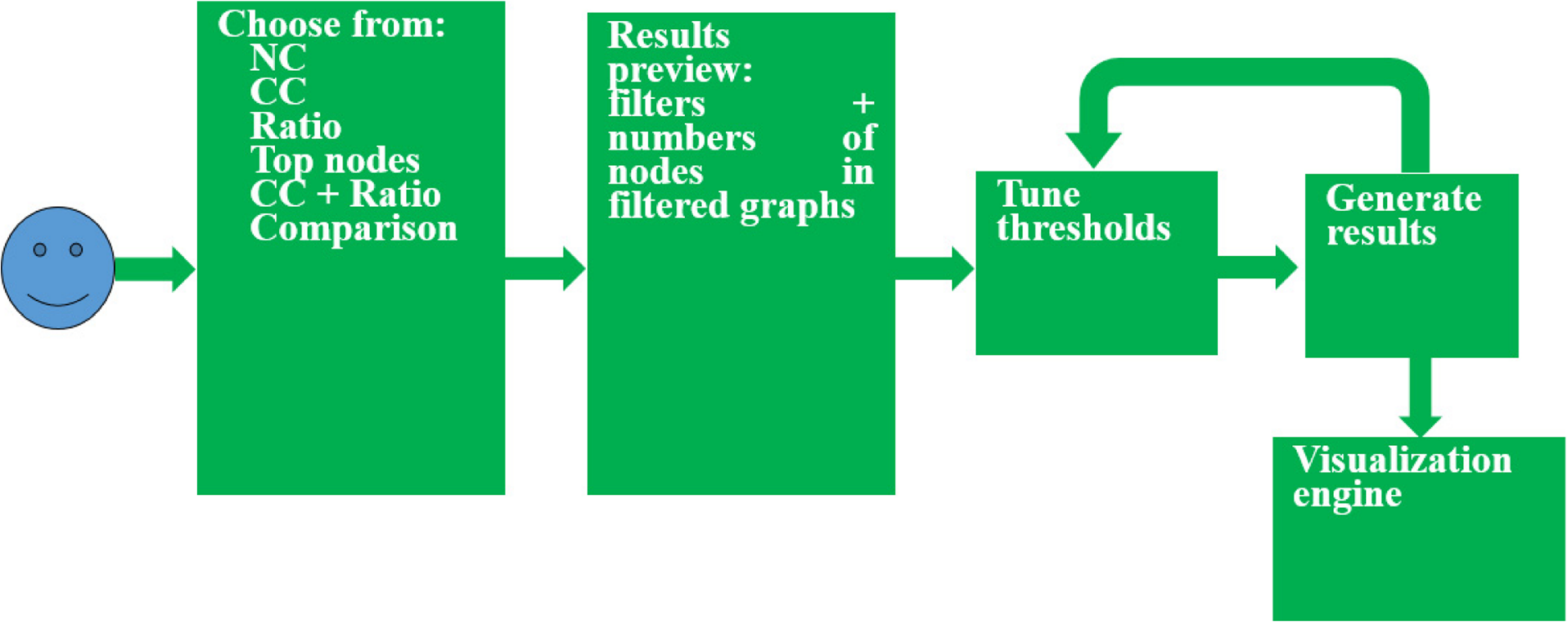
VIADS analytic engine workflow

The analysis module serves as the brain of VIADS. Figure 2 illustrates the workflow of VIADS’ analytic engine.

`

**Fig. 3:**
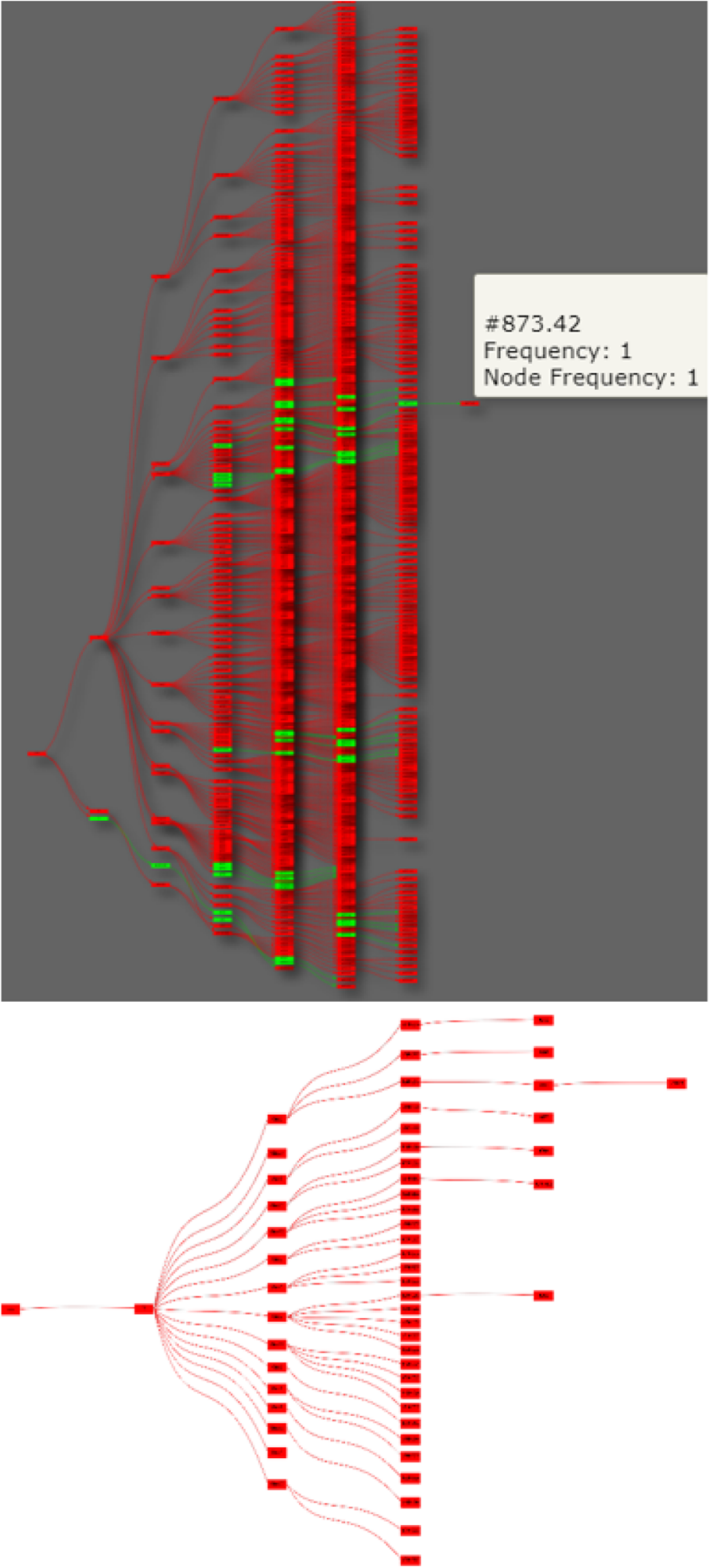
Graphs before (upper, an original graph with 1066 nodes) and after (lower, filtered graph with 56 nodes, top 5% CC) filtering within VIADS by using top CC% algorithm (colors indicate the values of CC; red > > green)

In the analysis module, users first select an algorithm. Then the results preview will facilitate users to make decisions on tuning thresholds. After users decide on the thresholds, the results will be generated and presented, and then the files (i.e., both graphs and data) can be downloaded for future usage. For registered users, the generated results will be saved for future use. The output files of VIADS are filtered graphs (PNG and SVG) and the corresponding data file (CSV). The input files that VIADS accepts are in CSV format with two columns, one for terminology codes and one for the frequencies of the terminology code. Figure 3 shows an example from VIADS: the two graphs generated by a test ICD9-CM data set before and after filtering. We selected the top nodes algorithm, top CC% to filter the data set. The upper graph is generated by the original data set with 1066 nodes; the lower graph displays the nodes that are top 5% of CC in the data set. The pop-up box, including extended information about each node, will appear if the mouse hovers on a node.

### Interactive visualization features in VIADS

Graphviz generates static graphs with limited customization options. VIADS graphs, in contrast, provide more interactive features, e.g., the capability to rearrange the graph using a physics simulation to ensure that all nodes are tightly packed together. In addition, the visualization module provides alphabetic node sorting, various spacing options between hierarchical levels, automatic resizing of the graphs, a pop-up box with extended information about the node when the user hovers over it, and color scales to reflect exact data behind the nodes and edges. The visualization module can fit 130 nodes easily and efficiently in an 11-in. laptop screen. With VIADS, users can drag and drop nodes, generate previews and images and increase or decrease node spacing with a few clicks. We anticipate that the additional interactive features will facilitate end-users’ comprehension of the filtered graphs. The comparative screenshots can be seen via this URL: https://www.viads.info/help/. User manual and video-audio tutorials are available within the web site to aid in using the tool.

## Discussion

### Comparison with similar tools

VIADS is at the intersection of data analysis, aggregating, filtering, summarizing, and visualization of medical terminologies. By nature, VIADS is a data analytic tool with visualization functions that is specialized for processing, aggregating, filtering, comparing, summarizing, and highlighting data sets coded by hierarchical terminologies. Current applications are only in the biomedical field, but the principles can be generalized to hierarchical terminologies in other fields. Although there is overlap between existing tools and VIADS. Other tools generally do not have the breadth of functionality, with respect to aggregating, summarizing, filtering, comparing, and visualizing, all of which are integrated into VIADS. During the earlier stage of this project, we developed the algorithms and conducted case studies [15] through the use of a variety of generic analytical tools, including internal pipeline, R, MS Excel, and MySQL, as well as through manual efforts in between. During the process, especially when we tried to share the detailed procedures with peers, we felt the need to provide a user-friendly, one-stop means that included ***all*** of the functions for users with modest programming experience. In this section, we compare VIADS with some of the other tools with similar functions.

Keylines [18] is a powerful *visualization* platform that provides analytic features and can be used for further development. The application fields of the analysis of Keylines, are focused on social media, information security networks, and pharmaceuticals. The nodes in their networks do not have close semantic relationships, as do the nodes within biomedical terminologies. Particularly, the pharmaceutical example has a different focus than that of VIADS. We recognize, however, that Keylines can be referenced for more sophisticated algorithm development and for advanced interactive features for VIADS.

neo4j [19] provides a powerful *management* platform for graph databases. Similar to Keylines, neo4j does not consider semantic relationships among nodes in the networks during analysis. neo4j and Linkurious [20] provide advanced capabilities to search nodes and edges, which do not exist in VIADS. VIADS, however, provides more algorithms to filter and to compare data sets based on calculations, statistical analysis, and semantic relationships within the terminologies. Visualizing results is only one module of VIADS; in addition, VIADS is an online, publicly accessible tool that can be used for educational and research purposes at no cost, in contrast to fee-based tools, e.g., KeyLines, neo4j enterprise edition.

There are also Gene Ontology visualization and analysis tools, such as AmiGo 2 [21] and GoMiner [22, 23]. GoMiner focuses on biological interpretations of omics data, such as microarray data. AmiGo is a search and browser tool for Gene Ontology and gene-associated products. VIADS, in comparison, is designed to be an analytic tool for phenotypes analysis and for the generation of summary views. The analytic results can be presented via the terminology structure in VIADS. Although both GoMiner and VIADS provide analytic capabilities, computation and statistical processes, and interactive visualization of results, they have different purposes and different applicable data sets. VIADS is a complementary analytic tool for non-genomics data. Table 4 provides a summary and comparison of the three tools. The comparison of the different tools, however, is meant to demonstrate the complementary roles of the tools, not imply that any specific one is better than another.

**Table 4:** Comparison of AmiGo 2, GoMiner, and VIADS

	AmiGo 2	GoMiner	VIADS
Analyze			
Provide biological interpretation	–	√	–
Upload data sets	–	√	√
Select algorithm/versions	–	√	√
Tune thresholds to filter	–	–	√
Generate summary views	–	√	√
Generate comparison views	–	–	√
Acceptable data set	–	Genomics data, Microarray data, proteomics data	Data sets coded with hierarchical terminologies + usage frequencies
Applicable terminologies	GO	GO	ICD9-CM, ICD10-CM, MeSH
Browse			
GO	√	√	–
Search			
External links	√	√	–
GO	√	√	–
Visualize			
Tree-like structure	√ (GO)	√ (GO)	√ (Summary views)
Statistical analysis results	–	√	√

During the algorithm development and VIADS design stages, we considered treemap [24] as a way to present the results. After much consideration, we decided to use our existing graphs to present the results. The main reasons are follows: (1) the existing graphs visually present our understanding of hierarchical terminologies in the medical field, based on our internal discussion; (2) the existing graphs can represent both poly-hierarchies and mono-hierarchies in a straightforward manner; and (3) the terminology structures used in VIADS include between 16,000 to 72,000 codes without filtering and over 100 codes with filtering. The layout of existing graphs provides a straightforward presentation in the scale that we need. It should be noted, however, that these are our design decisions. In the future, a well-designed and strictly controlled study of human comprehension of the graph we are using in VIADS and treemap may yield more reliable conclusions.

### Limitations, challenges, and lessons learned

Currently, VIADS supports only ICD9, ICD10, and MeSH. As noted, there are many more hierarchical terminologies in biomedicine that are not supported by VIADS. In the United States, ICD9 billing codes were replaced by ICD10 in 2015. To analyze longitudinal and historical data as well as to compare the data sets over time, supporting ICD9 is necessary. Because there are always updates in the terminologies, it is a challenge to maintain an accurate record of the terminologies year by year. More resources will be needed to build a stronger terminology service. Even in an ideal situation in which all terminologies were kept accurately on a yearly basis, if the users are not aware of the specific year of the terminology used for coding, the analysis results will not be ideal.

We have used a variety of libraries, programming languages, and frameworks in building VIADS. This presents a challenge in version control, system integration, and continuous deployment, especially when components are upgraded individually. The project team would have to monitor VIADS constantly to ensure that the production server is up and running in the correct work mode. If the server operation is interrupted, the project team will make adjustments accordingly.

We have not conducted a formal evaluation of VIADS. During the design, development, and revision stages, we did, however, include many team members’ input. Therefore, we cannot draw many user-related conclusions about VIADS. We can state only the functionalities without a detailed description of the level of facilitation that VIADS can provide. Meanwhile, we recognize that it is also necessary to compare different presentations of the hierarchies and their impacts in human comprehensions of the data sets.

Privacy is another concern of many potential users. We need to point out, however, that VIADS processes and analyzes aggregated data sets only in regard to the frequencies of different diseases (for ICD) or medical terms (for MeSH). In VIADS, there are no personal-level data. For aggregated frequencies, we also have strict criteria about the minimally acceptable sizes: Patient counts need to be equal to or greater than 100, and event counts equal to or greater than 1000, as listed in Table 2. Finally, although we recognize that there are no absolutely safe strategies on the Web, the risk of disclosure of individual patients’ data is relatively low.

### Mode of availability of software

VIADS is not an open source software. However, VIADS can be used for free for educational and research purposes. Any usage for commercial related purposes will need to contact the Ohio University Technology Transfer Office. All data sets uploaded or saved in VIADS belong to the original users. VIADS will not use any of these data sets for any other purposes but those for which VIADS is intended, with the exception of law enforcement requests under applicable laws. Routine website activities will be captured in log files for internal administration and reporting purposes.

### Future work

We are in the process of designing a usability study to formally evaluate VIADS and to improve the VIADS’ user experience. We will also conduct a utility study to explore how VIADS can be used to facilitate data-driven hypothesis generation among clinical researchers. In addition, a comparative user evaluation of different visualization representations and their impacts on human comprehension of the data set will be conducted.

VIADS users need to prepare their own data sets for analysis. We will post a data preparation document, including SQL queries, to guide users to prepare ICD9 and ICD10 data sets from their source databases. Currently, we encourage VIADS users to bring in their own data sets to process, analyze, and visualize. In the future, we will explore the possibilities of generating test data sets for education and training purposes. Another direction for future development is developing more sophisticated and advanced algorithms for VIADS data sets. Finally, in addition to supporting ICD9, ICD10, and MeSH, we plan to expand VIADS to support other hierarchical terminologies, such as LOINC.

Another future direction is to explore the possibility of incorporating VIADS as an analytic module in commercial EHR systems. The advantage of such an integration should be clear, as the analysis via VIADS would be conducted and updated in real time. This would be especially useful if a health department needs to aggregate results from different sources, as VIADS would allow tangible population health monitoring in real time.

## Conclusions

The rapid adoption of EHR systems in both office-based practices and hospitals has led to an increasing number of available coded data sets. These coded data sets are becoming increasingly common not only on the administrative side (e.g., for billing purposes) but also on the clinical side (e.g., to generate a problem list). The development of a publicly accessible tool will help users to achieve a summary view, secondary analysis, and visualization of their health data sets with minimal technical effort. VIADS shows more efficient screen space usage in a graph display and more interactive features. Being able to view and interpret larger health data sets with ease is a great advantage of VIADS. Furthermore, the interactive features provide a level of convenience that may facilitate users in interpreting the results. VIADS, along with all these features can be used to facilitate clinicians, clinical researchers, and healthcare administrators to make data-driven decisions more conveniently.

## Availability and requirements

**Project name:** A visual interactive analytic tool for filtering and summarizing large data sets coded with hierarchical terminologies (VIADS).

**Project homepage:** https://www.viads.info

**Operating system(s):** Any operating systems that can run Google Chrome or Firefox should work. We have tested VIADS in Windows 8 and 10, Mac’s OSX operating system, and Linux operating system Ubuntu 16.04.

**Programming language:** Python, JavaScript.

**Other requirements:** We have tested VIADS in Google Chrome and Firefox.

**Any restrictions to use by non-academics:** Any usage for commercial related purposes will need to contact the Ohio University Technology Transfer Office.

## Abbreviations

CC: Class counts
EHR: Electronic health record
GO: Gene ontology
ICD-10-CM: International classification of diseases 10th revision-clinical modification
ICD-9-CM: International classification of diseases 9th revision-clinical modification
LOINC: Logical observation identifiers names and codes
MeSH: Medical subject headings
NC: Node counts
VIADS: A visual interactive analytic tool for filtering and summarizing large data sets coded with hierarchical terminologies
